# Cellular models of Batten disease

**DOI:** 10.1016/j.bbadis.2019.165559

**Published:** 2020-09-01

**Authors:** Christopher J. Minnis, Christopher D. Thornton, Lorna M. FitzPatrick, Tristan R. McKay

**Affiliations:** a MRC Laboratory for Molecular Cell Biology, University College London, London WC1E 6BT, UK; b Centre for Bioscience, Manchester Metropolitan University, Manchester M1 5GD, UK; c Dept. Comparative Biomedical Sciences, Royal Veterinary College, Royal College Street, London NW1 0TU, UK

**Keywords:** Yeast, Batten disease, NCL, Ceroid, Cellular models, Neurodegeneration

## Abstract

The Neuronal Ceroid Lipofuscinoses (NCL), otherwise known as Batten disease, are a group of neurodegenerative diseases caused by mutations in 13 known genes. All except one NCL is autosomal recessive in inheritance, with similar aetiology and characterised by the accumulation of autofluorescent storage material in the lysosomes of cells. Age of onset and the rate of progression vary between the NCLs. They are collectively one of the most common lysosomal storage diseases, but the enigma remains of how genetically distinct diseases result in such remarkably similar pathogenesis. Much has been learnt from cellular studies about the function of the proteins encoded by the affected genes. Such research has utilised primitive unicellular models such as yeast and amoeba containing gene orthologues, cells derived from naturally occurring (sheep) and genetically engineered (mouse) animal models or patient-derived cells. Most recently, patient-derived induced pluripotent stem cell (iPSC) lines have been differentiated into neural cell-types to study molecular pathogenesis in the cells most profoundly affected by disease. Here, we review how cell models have informed much of the biochemical understanding of the NCLs and how more complex models are being used to further this understanding and potentially act as platforms for therapeutic efficacy studies in the future.

## Highlights

• Developments made in cellular models for neuronal ceroid lipofuscinosis (NCL) in basic biology and use as therapeutic platforms.
• Cellular models elucidating function of NCL proteins.
• NCL proteins implicated in the mTor signalling pathway.
• Patient-derived induced pluripotent stem cell (iPSC) lines have been differentiated into neural cell-types providing insights into the molecular pathogenesis of NCL.

## 1 Yeast models

Yeast models have been regarded as an exceptional experimental model system for all eukaryotic biology and carry many advantages to elucidating complex biological systems in the modern era [1,2]. Utilised for many decades, they possess all the classical eukaryotic organelles such as mitochondria, endoplasmic reticulum, vacuoles (equivalent to lysosomes), and Golgi apparatus, allowing significant insights into the mechanics and molecular cell biology of processes such as cell division, metabolism, trafficking, and autophagy [3].

There has been extensive research to understand both the budding yeast *Saccharomyces cerevisiae* and the fission yeast *Schizosaccharomyces pombe,* especially following the sequencing of their DNA in 1996 and 2002 respectively [4,5]. Their genomes were revealed in exceptional detail, which has led to a robust quantitative analysis of both the transcriptome and proteome profiles [6,7] as well as the development of techniques, that have allowed characterisations of the regulatory process and responses to environmental conditions [6]. *In-silico* techniques have also been utilised to predict and identify domains [8], including functionality. Additionally, both models have been used to develop metabolic profiles [9] as well as predict protein-protein interaction networks [10,11]. Impressively all this data can be accessed through their respective comprehensive open access genome databases, yeastgenome.org and pombase.org [12,13]. Furthermore, the synergy between valuable gene deletion libraries [14,15], robotic technologies and a wide range of functional genomic tools, provide a significantly robust platform to facilitate research in disease areas including neurodegeneration that affects fundamental cellular processes [16].

Both *S. cerevisiae* and *Sz. pombe* have been used to study the underlying cause of some neuronal ceroid lipofuscinoses (NCLs). The genes *CLN3*, *CLN10/CTSD* and *CLN12/ATP13A2* [17], are conserved in both yeasts, with *CLN1* also in *Sz. pombe* [18]. To date, most work using *S. cerevisiae* and *Sz. pombe* as models has focused on the orthologue of *CLN3* (*BTN1* and *btn1* respectively), which like CLN3, encode functionally ill-defined transmembrane proteins.

### 1.1 *CLN3*

Research carried out in both yeasts has been reviewed extensively [19], and will be summarised here. Within *S. cerevisiae*, BTN1 is known to localise to the vacuole and Golgi, with a role in the homeostasis of vacuolar morphology, cellular pH, storage and transport of amino acids [[20], [21], [22], [23], [24], [25], [26], [27]]. Similarly, in *Sz. pombe* Btn1 plays a far-reaching role in trafficking to the vacuole, regulation of vacuolar size and pH, Golgi morphology, cell morphology, cell polarity, cytoskeleton, temperature sensitivity, and osmotic homeostasis [[28], [29], [30], [31], [32], [33]].

Work in fission yeast has inferred that proteins carrying pathogenic mutations in *btn1* can retain different aspects of function [32]. Most relevant to juvenile CLN3 disease is the 1Kb deletion, which accounts for 81% of pathogenic mutations found in patients' [34], that results in a number of alternative transcripts [31]. A mutant protein equivalent to one of these transcripts in *Sz. pombe* exhibited partial functionality and was able to prevent some defects associated with complete loss of *btn1*, such as vacuolar size [31]. Other missense mutations within the luminal-facing domains cause significant effects on Btn1 function, most notably those affecting the amphipathic helix [32]. Whether there is a gain of function for some mutant CLN3 proteins is still under debate and is important in the context of the development of therapies for CLN3 disease and identification of therapeutic targets.

Genetic interactions are a good way of elucidating possible functions and identification of therapeutic targets [35]. A known genetic interaction between *BTN1* and *SDO1*, an orthologue of Shwachman-Bodian-Diamond syndrome (*SBDS*) in humans, was explored in *S. cerevisiae* using two-hybrid protein interactions, co-immunoprecipitation and co-localization [36]. SDO1 is a guanine nucleotide exchange factor (GEF) for ribosome assembly protein 1 (RIA1), another protein involved in ribosomal maturation predominantly located within the cytoplasm [37]. Absence of SDO1 leads to misregulation of BTN1 and a decrease in the ability of V-ATPase to pump protons but also made the vacuole more acidic. Although no direct physical interaction has been found between BTN1 and V-ATPase, it has been suggested that BTN1 negatively regulates ATP hydrolysis. It is unlikely that SDO1 and BTN1 directly interact with each other within the ribosomal maturation pathway. However, a link between CLN3 and RNA metabolism was suggested from work with a *Drosophila* model with enhanced CLN3 expression [38], with the suggestion that Sdo1p influences expression of BTN1 through a ribosomal monitoring network that affects vacuolar function and/or homeostasis. There has been increasing interest in the involvement of the ribosomal pathway in neurodegeneration, either through ribosomal stress granules [39], ribosomal stalling [40] or mRNA homeostasis [41].

High-throughput screening has helped in the understanding of global changes in the *Sz. pombe* model for *CLN3* disease, through investigating both the metabolome and genetic circuitry following the loss of Btn1 function [42,43]. Cells lacking Btn1 showed an increased glycolytic flux and TCA cycle, and the heat sensitivity phenotype was rescued *via* supplementation of glycolytic substrates, suggesting an adaptive response change through the glycolysis pathway [43]. Bond et al. took this adaptive change a step further, demonstrating a link between *btn1* and a conserved key signalling hub, the mTOR pathway [42]. The mTOR pathway is a highly conserved signalling network that coordinates multiple cellular functions from environmental cues [44]. It plays a fundamental role in cellular behaviour and physiology, particularly quiescence and longevity which are defective in *btn1*Δ cells [42,44]. Modulating TORC1 function genetically or *via* pharmacological inhibition was able to rescue all defects observed in the *Sz. pombe btn1*Δ model. This was also true following activation of the cell wall integrity (CWI) pathway by increasing the level of expression of *rho1*, reflecting the crosstalk between these two important pathways through Btn1 that may be inferred for CLN3 [42]. This finding is increasingly relevant as mTOR is emerging as a regulator of many neurological functions, such as circuit formation, neural control of feeding and neuronal development [45] as well as to the NCLs [46]. Deletion of mTORC1 or mTORC2 leads to reduced neuron size and early death [44]. Intriguingly, hyperactivation of the mTORC1 pathway, is a hallmark of Tuberous Sclerosis (TSC) in patients as well *Tsc1* or *Tsc2* knockout mouse models, that present with a range of neurological disorders including epilepsy, relevant to NCL pathology [47,48]. Both *Tsc1/2* and *Rhb1* (orthologue of *Rheb*) are conserved in *Sz. pombe*, but have not yet been fully investigated in relation to *btn1*. However, *Tsc1/2* and *Rheb* are not conserved in *S. cerevisiae* which may contribute to some of the mTOR associated differences between these models of disease.

Abnormal protein trafficking and membrane dynamics are a hallmark in many neurodegenerative diseases such as Parkinson's, Alzheimer's, Huntington's disease, as well as CLN3 disease [[49], [50], [51]]. First studied in *Drosophila* and mammalian systems, it was demonstrated that overexpression of CLN3 causes aggregation of Hook1, a regulator of endocytosis [52]. In *S. cerevisiae, BTN2* was assigned as a *Hook1* orthologue [52] and was upregulated in cells that lack *BTN1* [22]. Like Hook1, BTN2 was also shown to interact with SNARE complexes, binding with v-SNARE SNC1 [53]. Upon deletion of *BTN2,* retrieval of YIF1 transport protein from late endosomes to the Golgi apparatus was blocked. Similarly, BTN1 has been shown to regulate Golgi morphology *via* modulation of SNARE protein SED5 itself phosphorylated by YCK3 [22,54]. Golgi disruption is also observed in *Sz. pombe* upon the loss of *btn1*, with defects in Golgi morphology and location, which has been suggested to lead to dysregulation of the cell cycle and aberrant protein sorting of the vacuole hydrolase carboxypeptidase-Y [33]. In addition, *Sz. pombe* had defects in the polarisation of sterol domains, leading to loss of Myo1 localisation including defective formation of F-actin patches and disruption of the endocytic pathway [29].

Correct Golgi and sorting function are vital in both metabolism and distribution of lipid-bound membrane proteins. In *S. cerevisiae* BTN1 has a role in phospholipid distribution [55]. Absence of BTN1 leads to a decrease in phosphatidylethanolamine (PtdEtn) and phosphatidylserine (a precursor to PtdEtn) in the mitochondria and vacuolar membranes. *BTN1*Δ cells given ethanolamine (Etn) supplement did not rescue the PtdEtn concentrations and became toxic at higher doses. Since there was no observation of PtdEtn accumulation in the ER, it implies a dysfunctional Kennedy pathway [56] through the lack of substrate incorporation [55]. Therefore, the array of cellular phenotypes associated with the lack of BTN1, and by extrapolation CLN3, could be the consequence of altered phospholipid dynamics. Dobzinski et al. also observed links with TOR signalling pathways and the processing of Golgi proteins to vacuoles for degradation [57]. Inactivation of TOR under starvation causes Golgi-quality-control (GQC) substrates to be recognised by the vacuole protein machinery when ubiquitinated, through the mediation of the defective-for-SREBP-cleavage complex (DSC). Since the DSC complex in *Sz. pombe* promotes both ubiquitination and proteasome cleavage, it would be interesting to investigate a connection with MVB protein sorting pathway. The authors suggest that GQC substrate delivery upon starvation response may be a general pre-autophagic response essential for cell survival or quiescence from upregulation of MVB-VPS pathways.

The relevance of the regulatory link between BTN2 and BTN1 is yet not been fully elucidated. However the identification a third protein that is a negative regulator of BTN2, BTN3 (yeast orthologue of a human mitochondrial complex 1 deficiency gene) within the cellular stress response and prion curing, raises many interesting questions [58,59]. Current understanding is that BTN3 and SNC1 control BTN2 localisation and function. The observed inhibitory effects of prion curing suggested that BTN3 downregulates protein trafficking and prion aggregation from BTN2-mediated URE3, which may prove crucial during the onset of mitochondrial complex 1 deficiency and Batten disease in humans. BTN3 competes with SNC1 for BTN2's binding site, sequestering it from its substrates [58]. Equally, BTN3 has been shown to bind with ENT3 and ENT5 epsin proteins in late endosomes. This suggested that BTN3 regulates endosomal sorting function of adaptor proteins, BTN2, ENT3 and ENT5. Although one could argue that BTN2/3 will not help identify the function of BTN1/CLN3, these observations imply that the main pathway in Batten disease pathogenesis might be linked to defects in the endosomal recycling of proteins involved in vesicular fusion or to a yet to be identified prion-like substrate [60].

### 1.2 *CLN10*

*CLN10* (Cathepsin D) has functional orthologues in both *S. cerevisiae* and *Sz. pombe*, *PEP4* [61] and *Sxa1* [62], respectively. The *PEP4* model is well characterised, and in recent years it has become apparent that PEP4 is not solely a vacuolar degrader, which may offer insights into other dysfunctional pathways in *CLN10* mutations [63]. In *S. cerevisiae* PEP4 affects gene expression through formation of the SAGA-like (Spt-Ada-Gen5 acetyltransferase) or SLIK complex, having both an activation and inhibitory effect on genes [64,65]. PEP4 has also been shown to influence chronological ageing through anti-apoptotic and anti-necrotic processes. Intriguingly, delaying cell death is dependent on the PEP4's catalytic active state while the necrotic and survival activity in quiescent cells is conferred by PEP4's inactive state and is independent of PEP4's catalytic activation [66]. Additionally, overexpression of *PEP4* was beneficial in a Parkinson's disease (PD) yeast model, decreasing the accumulation of α-synuclein, that would normally trigger cell death by acidification [67]. PEP4 equalised pH homeostasis and vacuolar proteolytic functions, decreasing α-synuclein aggregates. Unexpectedly, this mechanism of action was dependent on functional calcineurin signalling leading to correct trafficking of PEP4 [67].

### 1.3 *CLN12*

The identification of *CLN12 (ATP13A2*) in a rare case of Juvenile NCL reminds of the relevance of other yeast models in NCLs and neurodegenerative disease [17,68]. CLN12 is part of the superfamily of ATPases transporting cations across cell membranes and is located on the late endosomal/lysosome with emerging evidence of it being a critical regulator of lysosomal function [[69], [70], [71], [72]]. Loss of function mutations in *ATP13A2* is the leading cause of Kufor-Rakeb syndrome (KRS), a rare form of early Parkinsonism [69]. *CLN12* has an orthologue in *S. cerevisiae, YPK9*. Although the molecular function and regulation of CLN12/YPK9 remains unclear, cells deleted for this gene were observed to have increased sensitivity to Cd^2+^, Mn^2+^, Ni^2+^ and Se^2+^ toxicity [73].

Similarly to *PEP4*, overexpression of *YPK9* was observed to reduce the accumulation of α-synuclein, a notable pathology in Parkinson's disease (PD) and also observed in CLN3 disease models [74,75]. This could infer an intrinsic link between the CLN12 and CLN3 patients who also exhibit Parkinson's-like movement disorder [76]. Chesi et al. characterised the *YPK9* network, using a genome-wide study and confirmed than YPK9 is essential for manganese homeostasis. Utilising this aspect, they observed YPK9 interacts with many proteins involved in trafficking and manganese homeostasis [75].

In *Sz. pombe CLN12/ATP13A2* has at least two homologues, *cta5* and *SPCC1672.11c*. Both are thought to encode for p-type ATPase transporters, with Cat5 also functioning at a homeostatic regulator for Ca^2+^ and Mn^2+^ [77]. Intriguingly, both yeast models of *CLN12* suggest in part CLN12 can act as a Mn^2+^ regulator; conversely, it is known that manganese is a risk factor for developing PD or PD-like syndrome, implying the importance of manganese homeostasis in both Batten and Parkinson's diseases [78].

## 2 *Dictyostelium discoideum*

The social amoeba has proven to be an excellent model organism to research neurodegenerative diseases, including NCL genes, (reviewed extensively in McLaren et al.) [79,80]. *Dictyostelium* encodes for 11 of the 13 known genes linked to NCL. This makes *Dictyostelium* an attractively simple model system to elucidate the function of NCL proteins [81] and provide new tools to explore NCL disease. Mutations in *CLN2(TPP1*) cause a late infantile form of NCL in CLN2 disease [76]. CLN2 is a serine protease that impacts fluidic phase endocytosis and autophagy [82]. In *Dictyostelium*, six genes share similarities with *CLN2*, and these are *tpp1A-F* [83]. Loss of *tpp1A* in *Dictyostelium* through homologous recombination leads to a decrease in overall Tpp1 activity and accumulation of storage material in cells that are starved [84]. Also, *tpp1A*^−/−^ cells are impaired in growth and viability when continuously grown in axenic autophagy-stimulating media (ASM) [82,84]. This suggested that Tpp1A is required for proper autophagic response under starvation conditions and is consistent with what was seen in patient fibroblasts which have reduced autophagosome formation [82]. Stumpf et al. have recently studied two other *TPP1* genes, *tpp1B* and *tpp1F*. Tpp1B/F is thought to bind to the Golgi pH regulator (GPHR) [83], an anion channel that regulates the morphology of both the Golgi complex and the ER [85,86]. The localisation of Tpp1F in the ER, Golgi complex, V-ATPase-positive vesicles and the binding of GPHR provide new insight into the potential implications of CLN2 binding to GPHR in human cell lines [83].

The *Dictyostelium* homolog of *CLN3* is *cln3*. Cln3 is also a transmembrane protein and localised predominantly to the contractile vacuole system but also the Golgi complex and endocytic pathway, consistent with other NCL models [[87], [88], [89]]. Cells lacking *cln3* display a variety of phenotypes during growth phase, including defects in cytokinesis and osmoregulation as well as increased cell proliferation [87,90]. During early development, *cln3*^−/−^ cells show delayed streaming and aggregation, impaired protein secretion and reduced adhesion [[87], [88], [89]].

Unlike yeast models, *Dictyostelium* also has a functional orthologue of *CLN5* [81]. Mutations in *CLN5* cause late infantile CLN5 disease [76]. In mammals, CLN5 is localised to the lysosome as well as being secreted [91,92]. Highlighting the conserved nature of the NCL pathways, cells lacking *cln5* in *Dictyostelium* also accumulate autofluorescent storage material [93]. The Cln5 interactome in *Dictyostelium* reveals that it interacts with a range of proteins including lysosomal enzymes, proteases and most notably includes other NCL proteins like TppB/Cln2, CtsD/Cln10 and CtsF/Cln13, in addition to proteins linked with Cln3 function such as quorum-sensing protein AprA and CadA, a calcium-dependent cell-cell adhesion protein [92]. This suggests that there is indeed an interconnectome between NCL genes, that have yet to be fully elucidated.

## 3 Mouse cell models

### 3.1 Cerebellar granule cells

Cerebellar granule cells (CGCs) have long been recognised as being a ubiquitously occurring within the mammalian brain [94]. First isolated from murine models for cell culture in the 1970s, the dissociation and growth of cerebellar granule cells have expedited the understanding of neuronal functionality in both wild-type and a disease-based context [95]. Several murine models recapitulating multiple *CLN* mutations have been produced from labs worldwide. From these models, CGCs can be isolated as primary cells or immortalised to study the molecular biology of disease. *In vitro* experiments using primary and immortalised CGCs from *Cln3*, *Cln6* and *Cln7* mouse models have been informative in better understanding NCL neuropathology.

There have been multiple *Cln3* mouse models produced. Mitchison et al. first described a *Cln3*^Δex1–6^ knockout murine model in 1999, followed by Katz et al. generating the *Cln3*^–/–^ knockout model and a *Cln3*^LacZ^ β-galactosidase reporter system being produced later [[96], [97], [98]]. A knock-in *Cln3* mouse model produced by the Cotman lab recapitulates the 1 Kb intragenic deletion that is the most common inherited mutation in patients' with juvenile CLN3 disease [99]. The resulting deletion removes exons 7 and 8 resulting in a protein that is predicted to be out of frame after 153 amino acid residues – with 28 unconventional amino acids existing at the C-terminus, although alternative splice variants bring the transcript back into frame. Previous studies provide conflicting perspectives as to the residual or null function of any CLN protein produced by mutated transcripts, or if the transcripts are even translated at all [31,[100], [101], [102]]. That said, analysis of the *Cln3*^Δex7/8^ homozygote mice revealed an absence of exon 7 and 8 transcription as well as variant transcripts which also lack exon 5 or retention of introns 1, 10 or 11 [103], suggesting alternative splicing leads to variant *Cln3* proteins being produced that are lacking different portions. Therefore, the isolation and immortalisation of CGCs from *Cln3*^Δex7/8^ murine models represented a foundation for insight into the pathomechanism of neuronal cell death in *Cln3*^Δex7/8^ mice.

Fossale et al. were the first to demonstrate an archetypal NCL phenotype within an immortalised CGC line isolated from *Cln3*^Δex7/8^ mice [49]. CGCs from the mutated mice (Cb*Cln3*^Δex7/8^) exhibit abnormalities in both mitochondrial and lysosomal size, distribution and function as well as the hallmark accumulation of ATPase subunit c, alongside reduced ATP levels and apoptosis. The Cotman lab also went on to produce an immortalised CGC line from *Cln6* knockout mice (CbC*ln6*^*nclf/nclf*^). They observed phenotypic similarities between the *Cln3* and *Cln6* mutated CGC lines but divergent transcriptomic profiling implying distinct functions in neurons converging on the same pathway(s) [104].

Primary CGCs cultured from the Cb*Cln3*^*Δ*ex7/8^ knock-in mice demonstrate an altered sensitivity and excitotoxicity in both AMPA & NMDA-type glutamate receptors *in vitro*. Abnormal cerebellar glutamate receptor function may result in motor deficits. Seven week old *Cln3*^Δex7/8^ knock-in mice were subsequently demonstrated to display a reduction in motor co-ordination – presenting both AMPA and NMDA glutamate receptor function as a potential therapeutic target [102,105,106].

In 2019, the Storch lab produced the first immortalised neuronal CGC line from *Cln7* knockout mice [107]. Several phenotypic abnormalities were described in relation to late endosome/lysosomal morphology, movement and distribution within CGCs. However, there was no conclusive evidence of defective autophagy in *Cln7*^*−/−*^ cells. From this, it was speculated that CLN7 might function in nutrient sensing and mTOR complex assembly. Moreover, it was suggested that the accumulation of protein aggregates in the brains of murine models might be a consequence of reduced axonal lysosomal migration. Yet, more work needs to be undertaken for the exact neuronal function of the CLN7 protein to be understood.

Finn et al., also highlights how mice with different genetic backgrounds may contribute to potential differences in phenotypes. Specifically, changes to glutamate receptor expression and excitotoxicity differed in cells isolated from mice of different genetic backgrounds, and this could therefore also influence how the receptor is dysregulated in a disease context [102].

### 3.2 Embryonic fibroblasts

Isolated between E12.5 and E17 into gestation, mouse embryonic fibroblasts (MEF) are relatively easy to culture and amplify for approximately 5–7 passages. There are a limited number of studies utilising MEFs isolated from *Cln3* null mice. However, in 2011, Getty et al. observed cytoskeletal, and migration defects in *Cln3*^−/−^ MEFs; myosin-IIB distribution in *Cln3* null cells was elongated indicating cytoskeletal abnormalities [108]. Wavre-Shapton et al. showed that MEFs isolated from *Cln3*^Δex1–6^ have disorganised membranes and reduced LAMP1 membrane recruitment [109].

In 2016, Brandenstein et al. isolated MEFs from *Cln*7^Δex2^ mice and immortalised them employing lentiviral overexpression of the oncoprotein SV40 large T antigen [110]. Pulse-chase experiments in these *Cln*7^Δex2^ MEFs revealed unaltered expression/maturation of cathepsin proteins (proteases found in the lysosome), implying no lysosomal acidification defect in *Cln7* ^Δex2^ MEFs [110]. These data contrast with *in vivo* studies and data obtained from cultured neurons from *Cln3*^Δex7/8^ [49] and *Cln1* [111], which both have impaired trafficking and altered maturation of lysosomal enzymes. However, a lack of consistency in phenotype could be due to the cell-type evaluated rather than disease.

A recent study described a role for Cln7 in lipid metabolism in lysosomes in immortalised MEF cultures. In 2018, Danyukova et al. observed functional pathology in the *Cln*7^Δex2^ immortalised MEFs; the ability to adapt to starvation was impaired, and mTORC1 activity was defective. Furthermore, an increase in perinuclear accumulation of enlarged lysosomes was noted [46]. The group also performed SILAC-based quantitative mass spectrometry analysis that revealed a significantly different enzyme profile in the lysosomes of wild-type (WT) and *Cln7* KO MEFs. Notably, the soluble enzyme Cln5 (linked to late infantile NCL disorder) was significantly reduced in the *Cln7* KO lysosomes. This study is the first of its kind to describe the lysosomal proteome in NCL cells.

### 3.3 Neural/glial co-cultures

Although *in vitro* models have substantially contributed to the knowledge of the mechanisms of NCL, monolayer single cell type cultures cannot fully represent the complexity of NCL pathogenesis in the brain.

Interest in the contribution of glia to the initiation and progression of neurodegenerative diseases has risen in recent years. Two studies to date have isolated mixed glia and neurons from Batten disease mice and analysed cellular function in both co-culture and isolated cultures [112,113].

Astrocytes and microglia from CLN3 deficient mice (*Cln3*^*Δex1–6*^*)* displayed an attenuated ability to activate in response to stimulation alongside a modified protein secretion profile [113]. Moreover, the co-culture of diseased neurons and PPT1 diseased astrocytes resulted in exacerbated neuronal pathology. Conversely, co-culturing disease neurons with healthy glial promoted neuronal survival; this indicates that astrocytes may play a large role in neuron survival in Batten disease onset and progression. There is a similar input of glia into the disease progression of neurons cultured from *Ppt1*^*−*/−^ (*CLN1*) mice; when diseased neurons were co-cultured with defective glia, the neuronal phenotype was again significantly accelerated, and neuronal death was elevated [112]. Likewise, when cultured with healthy glia, the phenotype observed in neurons was ameliorated. When cultured individually, astrocytes and microglia displayed a more activated phenotype than healthy cells. Of note, *Ppt1*^−/−^ astrocytes display abnormal calcium signalling and increased cytoplasmic Ca^2+^ levels. Moreover, cell survival rates were decreased.

Considering that glial cells play a role in glutamate clearance in conjunction with oxidative stress reduction in neurons, it is perhaps not unsurprising that increased neuronal death occurs upon co-culture. These models could prove to be useful in future as they have shown that they recapitulate a phenotype observed *in vivo;* depletion of reactive astrocytes in *Ppt1*^−/−^ mice result in exacerbation of neuropathology [114]. In *Cln3*^*Δex7/8*^ mouse astrocytes, Ca^2+^ signalling was attenuated, and astrocyte metabolism is dysregulated despite normal levels of mitochondria. Consequently, *Cln3*^*Δex7/8*^ neurons respond in a hyper-sensitive manner indicates the significance of cell-cell interactions when modelling NCL and other neurodegenerative disease [115].

### 3.4 Blood brain barrier cell models

Within the central nervous system (CNS), *Cln3* is expressed in the vascular endothelium as well as in neuronal and glial cells [96,116]. Correspondingly, abnormalities in the blood brain barrier (BBB) are detectable in CLN3 patients. Two studies have shown autoantibodies against CNS proteins are present in CLN3 patients' blood and mouse models [117,118], suggesting that the BBB is compromised. A primary cell mouse model of the BBB [119] mouse brain endothelial cells (mBECs) express markers of adheren junctions (VE-cadherin and beta-catenin) and tight junctions (claudin-5, occlusion and ZO1).

Tecedor et al. isolated and immortalised mBECs from *Cln3*^+/+^ and *Cln3*^*lacz/lacz*^ knock-in mice [120]. This study showed that CLN3 is critical for normal caveolae-dependent endocytosis *via* Caveolin (Cav 1), an essential scaffolding protein. *Cln3*^−/−^ cells demonstrate abnormal distribution of (Cav) in the plasma membrane suggesting a role for CLN3 in anterograde trafficking of the protein. Moreover, this study showed CLN3 alters the composition of the plasma membrane and the trans-Golgi network and that drug reflux and cell volume modulation is impaired when CLN3 is lost, consistent with yeast studies [120]. In 2014, Schultz et al. showed that fluid-phase endocytosis is also impaired in the immortalised mBECs as determined by dextran uptake [121]. CLN3 may play a role in the deregulation of the Cdc42 activation pathway which modulates synthesis and breakdown of actin following fluid-phase uptake. Cao et al. also showed that fluid-phase endocytosis was impaired in both *Cln3*^Δex7/8^ and *Cln6* ^nclf/nclf^ cerebellar cells, suggesting similar roles for NCL proteins in fluid-phase endocytosis across cellular models of Batten disease [49,52,104,121].

In a follow-on study carbenoxolone (CBX) was identified, as a potential therapeutic agent for CLN3 patients. CBX, a drug that blocks hemichannels, was shown to alter lipid microdomains, normalise Cav1 signal at the plasma membrane and improve the membrane fluidity alterations observed in *Cln3*^−/−^ mBECs [122]. Moreover, CBX improves Cdc42 signalling defects in *Cln3* null mBECs. Although the mechanism is not fully understood, CBX is known to stabilise lysosomal membranes and has shown to improve some pathological effects in *Cln3*^Δex7/8^ mice [123,124]. CBX cannot cross the BBB; thus, its metabolite, enoxolone, is most probably causing the therapeutic effect.

In summary, the murine mBEC *Cln3*^−/−^ cells display a phenotype corroborated in cerebellar cells, the models have elucidated further understanding of a mechanism of action of CLN3 in endothelial cells, and a potential therapy could be explored. Although mBECs do not display a build-up lipofuscin, this might be due to cell proliferation. However, the BBB is a complex structure, where astrocyte cell projections (“feet”) surround and provide biochemical support [125]; complex cell models may be required to elucidate further the role of NCL proteins in BBB integrity.

### 3.5 Induced pluripotent stem cells (iPSC)

iPSCs have been generated from wild-type and *Cln1/5* double knockout (C*ln1*/5^ko/ko^) mice and their differentiation capacity compared with wild-type. Embryoid bodies (EB) differentiated from *Cln1/*5^ko/ko^ iPSC were smaller, abnormally shaped and with less differentiated cells when compared to controls implying delays in differentiation capacity, although no detail on cell-type distribution was provided. These data could imply developmental impedance but require much more detailed evaluations [126].

## 4 Sheep cell models

Work has utilised neurons, isolated and cultured from natural sheep models of CLN5 and CLN6 disease for ovine ceroid lipofuscinosis (OCL) [127]. Overall, the body of work largely provided early evidence of the accumulation of autofluorescent storage material alongside the accrual of ATPase subunit c [[127], [128], [129]]. The reconstitution of *CLN5* expression also resulted in the reversal of the prolonged storage of autofluorescent material and ATPase subunit c within the ovine neurons. Recently, the ovine model is being utilised for pre-clinical evaluations in *CLN5* gene therapy trials as a large mammalian model, where *in vitro* cell models are sure to play a significant role in establishing efficacy [130].

## 5 Human cell models

Much of the sub-cellular localisation and molecular biology of the NCLs have been elucidated using human cell models, from generic immortalised cell lines to primary and immortalised cell lines derived from patients and most recently patient-derived induced pluripotent stem cell (iPSC) lines. The vast majority of studies relate to late infantile CLN2 disease and juvenile CLN3 disease, although there are some mechanistic studies of infantile CLN1 disease.

### 5.1 Primary fibroblasts

While standard cell lines have provided a cellular base to study the ectopic expression of *CLN3*, primary cells derived from patients with NCLs give context to null or aberrant CLN protein expression. Dermal fibroblasts have been widely utilised because skin biopsy has historically been included for biochemical confirmation of disease. However, data must be contextualised in that there is no gross Batten disease pathology in the skin. Nevertheless, some of the earliest biochemical studies utilised patient fibroblasts to identify defects in lysosomal activity and substrate accumulation. For example Bennett et al. first described defects in lysosomal cathepsin activity in late-infantile and juvenile NCL fibroblasts [131]. The ATPase subunit c in lysosomal substrate accumulation is described in CLN2 [132,133] and CLN3 [134] disease fibroblasts, and has been related to cellular senescence [135] abnormalities in fatty acid oxidation [136] and lysosomal macroautophagy [133]. More recently, Na^+^/K^+^-ATPase turnover at the plasma membrane is shown to be disturbed through disruption of CLN3 interaction with Fodrin, which promotes endocytosis of the ion channel [137].

### 5.2 Lymphoblasts

Patient-derived lymphoblast cells have either been employed as primary cultures or more often immortalised to generate cell lines, for study. Although like fibroblasts, peripheral blood derived lymphoblasts are not directly affected by NCL disease, they are relatively easy to source and isolate ethically. Early evaluation of lipofuscin has been performed in CLN1, CLN2 and CLN3 patient lymphoblasts [138]. Multiple studies have described an increased sensitivity to apoptotic stimuli in immortalised *CLN3* lymphoblasts when compared to controls. Chemotherapy-induced apoptosis has been shown to be stabilised by reconstitution of wild-type *CLN3* expression [139], resveratrol-mediated reduction in ROS and ER stress [140] and fibrate treatment maintaining mitochondrial integrity and macroautophagy [141]. Dhar et al. observed that neurons in *CLN2* and *CLN3* disease could survive through the upregulation of Bcl-2, a neuroprotective molecule. Furthermore, they showed that Flupirtine (a triaminopyridine derivative that upregulates Bcl-2) was able to rescue etoposide-induced apoptosis in *CLN1, CLN2, CLN3 and CLN6* deficient lymphoblasts [142]. Flupirtine could be useful in prevention or halting the progression of NCL disease, however more research needs to done to confirm the underlying mechanism of action.

### 5.3 Neural & glial cells

NCL genes are ubiquitously expressed throughout the body, however cells of the CNS show heightened sensitivity from the effects of Batten disease, and currently, it is not feasible to study such patient cells in the laboratory. Alternative approaches to studying NCLs in relevant cells are to obtain healthy donor CNS cells (although these are derived from adult tissue whereas the disease is predominantly seen in childhood, raising ethical issues) or to exploit new technologies and generate these cell types from patient-derived induced pluripotent stem cells (iPSC) [143]. Ghosh et al. tested the efficacy of upregulating *TPP1* expression as a therapeutic approach for late infantile CLN2 disease, where many causative mutations can result in low-level expression, by pharmacologically agonising the PPARalpha/RXRalpha pathway [144]. They used fetal brain tissue from the Human Embryology Laboratory (University of Washington, Seattle, USA) and purified astroglia through long-term culture. These cells expressed functional TPP1, which could be upregulated by the application of fibrate drugs and all-trans retinoic acid.

The emergence of iPSC technologies provides a unique ability to study disease abrogation in a personalised medicine context. Archived skin fibroblasts or peripheral blood can be used as starting material to generate iPSC lines which can then be differentiated into neural cell types to study the underlying biology of disease or, critically, responses to potential therapies. To date, there have been multiple models for NCLs derived from iPSC [[145], [146], [147], [148]]. Chandrchud et al. used *CLN3* iPSC-derived neural progenitor cells (NPCs) as a follow-up to their screening platform, further evaluating potential therapies for the treatment of neuropathology [149]. They employed a novel biochemical assay, screening against the accumulation of the autophagy protein LC3 by constitutively expressing a GFP-LC3 fusion. Thapsigargin, an ER Ca^2+^-ATPase inhibitor, was identified as a drug capable of increasing GFP-LC3 puncta in *CLN3*^*Ivs13/E15*^ and *CLN3*^*homΔex7/8*^ iPSC-derived NPCs.

Similarly, Sima et al., generated three neural stem cell lines from three patient iPSC lines, one for *CLN1* and two for *CLN2.* Importantly, they exhibited characteristic hallmarks of the disease phenotypes such as enlarged lysosomes, lipid droplet accumulation and storage material of ATPase subunit c. What was unique about their approach was that they used this model with replacement therapy and δ-tocopherol (DT), which was able to rescue the phenotypes. Additionally, they were able to partially ameliorate the phenotype further with the additional use of hydroxypropyl-β-cyclodextrin (HPBCD) [148]. This represents an important step for the use of these models for pathophysiology and drug development.

Uusi-Rauva et al., 2017 generated *CLN5*^*Y392X*^ iPSC lines that had the most common *CLN5* mutation [146]. These cells mimicked many of the cellular hallmarks seen in other NCL models as well as *CLN5* specific models such as accumulation of autofluorescent material, and disturbances in the lysosomal structure as seen in neuronal cells generated from *CLN3* iPSCs [145,146]. These cells will require further studies to clarify the exact role of CLN5 and CLN3 within the endosomal/lysosomal systems, particularly with their common interactor protein RAB7 [150,151].

The potential for the application of NCL patient-derived iPSC in discovery and therapy research is vast. The New York Stem Cell Foundation (NYSCF; https://nyscf.org) in collaboration with the Beyond Batten Disease Foundation recently announced the availability of multiple *CLN3* iPSC lines with family member controls. Equally, the Human IPSC Initiative (HipSci; http://www.hipsci.org) in the UK has generated a series of Batten disease iPSC lines spanning many genetic forms of the disease. Each repository is available to academic researchers worldwide.

However, with these recent advancements the lack of specific antibodies that recognise NCL proteins for different species remains a challenge to overcome [152].

## Transparency document

Transparency document Image 1
